# Comparative Effectiveness of Titanium Platelet-Rich Fibrin and Connective Tissue Graft Harvested from the Tuberosity Area Via Modified Vestibular Incision Supraperiosteal Tunnel Access for Managing Gingival Recession: Protocol for a Randomized Controlled Trial

**DOI:** 10.2196/67168

**Published:** 2025-10-02

**Authors:** Sanehi Punse, Prasad Dhadse

**Affiliations:** 1 Department of Periodontics and Implantology Datta Meghe Institute of Higher Education and Research (DU), Sawangi (M) Maharashtra India

**Keywords:** modified vestibular incision supraperiosteal tunnel access, M-VISTA, titanium-prepared platelet-rich fibrin, T-PRF, gingival recession, maxillary tuberosity, connective tissue graft, periodontal surgery, periodontist, periodontology, dentistry, gum, gingivitis, gingival recession, graft, gum graft, randomized controlled trial, RCT, protocol

## Abstract

**Background:**

Periodontal surgery has traditionally relied on connective tissue grafts (CTGs) obtained from the tuberosity site to correct gingival recession abnormalities. However, there are challenges to be addressed, including insufficient graft quantity and patient susceptibility. Consequently, titanium-prepared platelet-rich fibrin, or T-PRF, has emerged as a competitive alternative. By combining T-PRF with the modified vestibular incision supraperiosteal tunnel access (M-VISTA) approach, a more conservative way is provided, which may improve the course of treatment.

**Objective:**

The objective of this study is to compare the effectiveness of T-PRF and CTGs from the tuberosity area in managing gingival recession defects using the M-VISTA technique. The study aims to evaluate improvements in clinical outcomes, including pocket probing depth (PPD), clinical attachment level (CAL), relative gingival margin level (RGML), recession depth (RD), and width of keratinized gingiva (WKG), as well as plaque and bleeding indices.

**Methods:**

The proposed methodology entails conducting a randomized clinical trial over 2 years with 24 participants, each presenting with multiple gingival recessions (>2 mm, Miller’s Class I or II) on the buccal or labial aspects of teeth in the maxilla or mandible. Participants will be randomly allocated into 2 groups: the test group and the control group. The M-VISTA technique will be used for root coverage using T-PRF in the test group and tuberosity CTG in the control group as regenerative materials. Their effectiveness will be compared by evaluating PPD, CAL, RGML, RD, and WKG. Additionally, the plaque index will be calculated by dividing the total plaque index score of all teeth by the number of teeth examined, and the papillary bleeding index will be assessed using a periodontal probe with scores evaluated on a scale of 0-4 based on bleeding potential. Data will be analyzed using Student paired and unpaired t tests to compare results from baseline to 3 and 6 months for each group.

**Results:**

Recruitment, participant selection, baseline data collection, and randomization of groups concluded in September 2024, and the intervention phase is scheduled to end in December 2025. The study is expected to be completed by July 2026, with final evaluation, data analysis, and publication preparation taking place between June and July 2026.

**Conclusions:**

Based on existing evidence, we anticipate that the M-VISTA technique with T-PRF will provide superior root coverage compared to tuberosity CTG because no second surgical site is involved. Evidence suggests that T-PRF may offer comparable clinical benefits to CTG, particularly in clinical attachment gain, RD reduction, and gingival thickness, supporting the advancement of minimally invasive periodontal plastic surgery.

**Trial Registration:**

Clinical Trials Registry of India CTRI/2024/07/071619; https://tinyurl.com/ypfe34b5

**International Registered Report Identifier (IRRID):**

DERR1-10.2196/67168

## Introduction

The objective of periodontal treatment is to reestablish the supporting tissues destroyed by periodontitis and anticipate the progression of the disease itself. Aesthetics are another imperative matter that must be considered [1]. Gingival recession (GR) occurs when the apical gingival margin tissue migrates towards the cementoenamel junction (CEJ), leading to the root surface being exposed [2]. Many epidemiological studies have revealed that receding gums is a common condition in day-to-day clinical practice. The frequency of the disease fluctuates between 40% and 100% based on the population and analysis methods [1,2].

Several approaches have been made to manage GR, such as the lateral sliding flap, the double papilla flap, the envelope technique, and graft materials used in conjunction with advanced flap techniques [3]. Recent developments have led to the use of grafting techniques to upgrade the thickness and width of exposed tissue and to cover roots or implant surfaces [4]. The decision to proceed with the procedure is influenced by factors such as recession depth, attached gingiva width, number of teeth, and color balance after surgery. Each technique has its own distinct signs and constraints.

Using the envelope flap method, Raetze in 1985 [5] was the first to use this technique to cover individual GR. As described by Allen in 1994 [6], this method was developed to treat several GRs and later it was described by Zabalegui et al in 1999 [7] as the “tunnel” approach. However, the disadvantage of this technique was entry from a small sulcular point, which increased the possibility of damage and penetration of sulcular tissues, producing unsatisfactory outcomes. Because of these constraints, the vestibular incision supraperiosteal tunnelling access (VISTA) technique was developed by Zadeh [8], as a means of avoiding the potential complications associated with some tunnelling techniques. Several graft materials have been tested with VISTA and proven to be effective.

Modified VISTA (M-VISTA) is a modern and minimally invasive technique used for both generalized and localized GR defects [9]. This technique is particularly useful as it allows easier access to the vestibule by requiring a single vestibular incision that provides entry to the surrounding area. This permits adequate visualization of root and bone structure as well as dehiscence. It facilitates wound repair by reducing the complexity of the tunnelling process [2].

The paradigm for root coverage in periodontal surgery is autologous connective tissue graft (CTG). Traditionally, intraoral donor sites have been found in tuberosity or the palate for soft tissue grafts. Maxillary tuberosity block bone grafting was first described by Tolstunov in 2009 [10]. The evidence that tuberosity has more connective tissue fibers than adipose and glandular tissue and can shrink less is what led to its selection as a donor site [11]. The connective tissue covering the tuberosity contains dense collagen fibers with a well-keratinized epithelial layer. Only CTGs increased buccal gingival thickness after recession coverage, and residual fatty and glandular tissue could potentially impede plasma circulation and revascularization in early healing [12].

However, the thickness or quantity of CTGs may not be adequate for treating multiple GRs or donor area dehiscence in patients with thin palates. Additionally, when a patient refuses surgery twice, CTG is unfavorable [13]. Nonautogenous materials, like acellular dermal matrix and enamel matrix proteins, have applications in treating GR and as a substitute, but they cannot replace gold standards.

Platelet-rich fibrin (PRF) is a concentrated blood product selected for the fixation and revascularization of grafts and flaps because of the growth components it contains. A glass or plastic tube is obscured with activated silica from which a blood sample is extracted, which then generates into a fibrin [14]. Other scientists have conjectured that these particles of silica, which may not be present in the material, act as a catalyst and could potentially harm patients. To overcome the restraints of PRF, titanium-prepared PRF (T-PRF) emerged. Compared to PRF, the fibrin network of T-PRF is more compact as well as bulky. This compact structure of fibrin is important for promoting the retention and recruitment of growth factors into tissue over an extended period [15].

This randomized clinical trial protocol aims to evaluate the effectiveness of T-PRF and CTGs harvested from the tuberosity area using M-VISTA for the management of human GR defects with regards to root coverage (RC), gain in clinical attachment level (CAL), gingival thickness (GT), and improvement in the width of keratinized gingiva (WKG).

## Methods

### Overview

This study is a randomized clinical trial and will be performed over a period of 2 years. After obtaining written informed consent, the study will be carried out on 24 participants who will have multiple GRs (>2mm, teeth presenting Miller class I or II areas) on the buccal or labial regions of teeth in the maxilla or mandible from the outpatient department of Periodontics and Implantology at Sharad Pawar Dental College, Sawangi (Meghe), Wardha, Maharashtra, India. T-PRF clot (test group) and tuberosity CTG (control group) will be the biomaterials used in the M-VISTA procedure for root coverage, and their efficacy will be contrasted.

### Ethical Considerations

The Institutional Ethics Committee Datta Meghe Institute of Higher Education And Research, Sawangi (Meghe), Maharashtra, India, has reviewed and given approval to the study protocol, guaranteeing adherence to moral principles and norms (DMIHER(DU)/IEC/2024/05). This confirmation attests to the fact that participant safety, confidentiality, and informed consent will all be taken into account during the study’s execution.

### Sample Size Calculation

The sample size calculation was based on the formula for determining the required sample size when comparing 2 means, where the outcome of interest is the difference in clinical measurements (such as probing pocket depth [PPD] and CAL) between 2 groups (T-PRF and CTG). The data were evaluated from the earlier research of Collins et al [16] by using the SPSS 27.0 version (IBM Corporation) open-source calculator to determine the sum of squares mean. The outcome of the evaluation was 12. Thus, 12 participants will be unmethodically divided into the 2 groups.

The formula used was:




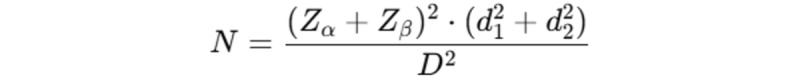




Where Z_α_ is the Z-value corresponding to the significance level (α), which is typically 0.05 for a 95% confidence level; Z_β_ is the Z-value corresponding to the desired power (β), which is typically 0.20 for 80% power; d_1_ and d_2_ are the SDs of the 2 groups being compared (T-PRF and CTG); and D is the expected difference between the 2 means.

Given Z_α_=1.96 (for a 95% confidence level), Z_β_ =0.84 (for 80% power), SD = (1.32 + 1.10) / 2, and D=1.07 (representing the clinically significant difference between the 2 treatment groups), the sample size calculation is as follows:




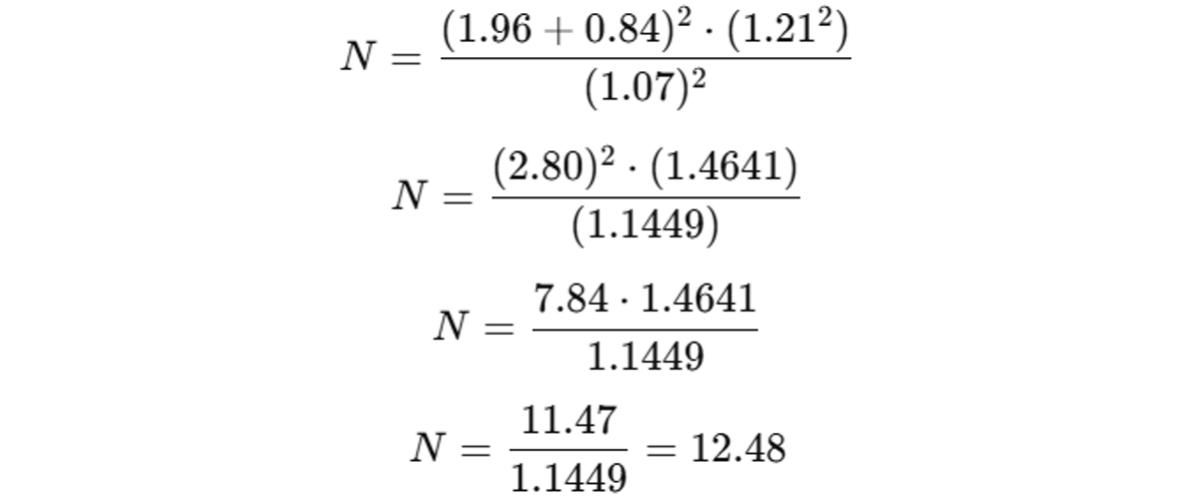




Thus, the sample size required per group is approximately 12 participants. Since the result is rounded, the final sample size is set to 12 per group.

Randomization ensures that each participant has an equal probability of being assigned to either group, thereby preventing systematic differences between the treatment arms that could influence the outcomes. The random allocation of 12 participants per group was implemented to minimize selection bias and enhance the internal validity of the study using a computer-generated randomization sequence to minimize selection bias and enhance the study’s internal validity.

### Inclusion Criteria

This study included systemically healthy patients who (1) were nonsmokers, (2) were aged between 18 and 65 years, (3) had teeth presenting Miller class I or II areas with multiple GRs on the buccal or labial surfaces present in either the maxilla or mandible, (4) had a GR depth of ≥2 mm, and (5) had X-rays of adequate interdental bone (separation across crestal bone and CEJ ≤ 2 mm).

### Exclusion Criteria

The following patients were excluded: patients using tobacco products, uncooperative patients, individuals with inadequate oral hygiene and a plaque score ≥1 after nonsurgical treatment, individuals with a history of periodontal surgical treatment in the selected research area, pregnant or lactating women, patients with mobile or severely decayed teeth, and those with irregularly placed teeth. Additionally, patients with systemic conditions such as hematological disorders (eg, anemia, thrombocytopenia, or clotting factor deficiencies), those undergoing anticoagulant therapy, or individuals with chronic kidney disease, uncontrolled diabetes mellitus, liver diseases (eg, cirrhosis), and severe cardiovascular conditions requiring anticoagulant therapy were excluded to ensure the study’s integrity and minimize complications.

### Procedure

The process of scaling, root planing, and polishing will be done on every patient. The patient will be instructed to use the modified Stillman brushing method to minimize brush injury. Unless the patients receive a plaque score ≤1, oral hygiene instructions will be provided. A review will be conducted 6 weeks later to evaluate plaque control and tissue response. The patient’s participation in the study is contingent on their poor oral hygiene. The patient will provide with informed consent after being given information about the study plan.

### Clinical Measurements

#### Indices

All patients’ oral hygiene and gingival status are reviewed on the day of surgery, 3 and 6 months apart.

Plaque index (PI) [17]: The presence of plaque is evaluated on the labial or buccal and lingual surfaces of all teeth after the application of the disclosing agent (Table 1). The PI value for the tooth is calculated by splitting all the points around it by 2. The PI score for each individual person is gained by summing the PI scores of all teeth and dividing by the number of teeth inspected.

Papillary bleeding index [18]: The exact mesial introduction of a periodontal probe (William’s Calibrated Probe) into the gingival sulcus at the papilla’s apex will be followed by a coronal advancement to the tip of the papilla. On the same papilla, this is repeated on the distal side. On a scale of 0-4, the degree of potential bleeding is evaluated. The papillary bleeding index score per individual will be calculated by adding up all papillary bleeding index scores to the number of surfaces analyzed.

**Table 1:** Plaque index.

Score	Criteria
0	Absence of plaque.
1	Distinct plaque deposits on the cervical surface of a tooth.
2	Thin, uninterrupted layer of plaque (up to 1 mm) covering the tooth’s cervical margin.
3	Plaque band broader than 1 mm but covering less than one-third of the crown.
4	Plaque covering not less than one-third but less than two-thirds of the crown.
5	Plaque that covers two-thirds or more of the crown.

#### Clinical parameters

Clinical parameters measured for outcome assessment are relative PPD values, attachment level, relative gingival margin level (RGML), recession depth (RD), WKG, and GT value noted with an UNC-15 probe. An acrylic cast will be made for the patient to obtain clinical parameters. An acrylic stent will be used  to cover the occlusal regions of the test tooth. By directing it using burs, the periodontal probe extends into the deepest defect site. The stent, which will act as a guide for the periodontal probe, will develop longitudinal patterns. Under local anesthesia, the GT will be measured 3 mm below using a rubber stopper and endodontic reamer to assist the gingival edge. From the cementoenamel junction to the gingival margin, RD will be evaluated. WKG will be reached by calculation of sulcus depth and attached gingiva with the UNC-15 probe. These treated areas will be measured clinically on the day of surgery and after 3 and 6 months.

#### Radiographic Measurement

Grid specifications and intraoral periapical assessment will be used to show that there is enough interdental bone.

### Preparation of the T-PRF Membrane

Venipuncture, which entails taking a 10-mL blood sample from the antecubital vein, is used in the manufacture of T-PRF. It undergoes a single spin centrifugation at 2700 rpm for 12 minutes. Following centrifugation, a T-PRF clot will be produced. The fibrin clot that has formed inside the tube will be removed together with the remaining red blood cells [14,15].

### Surgical Procedure

#### Surgical Procedure for the Test Group

Before the treatment, patients will be instructed to cleanse their mouth using 0.2% chlorhexidine gluconate for 1 minute. The surgical method is focused on preventing infection and maintaining a flawless aseptic approach. Following initiation of 2% local anesthesia, the exposed root areas are precisely planned using an ultrasonic device and then with curettes. When injected with local anesthesia, all intracrevicular incisions will be given using a microsurgical blade, with the exception of inferior incisor incisions, which will be made with a smaller, disposable blade. A full-thickness, 8-10 mm, vertical incision shall be created in the vestibule, either mesial or distal to the surgical location being operated on. This incision (vertical incision) will be limited to the gingival margin and will serve as an entrance for the widening of the supraperiosteal tunnel. Given the length of the teeth to be cured, a vertical incision will be created especially in the central area. The incisions will extend not less than 1 tooth over the ones to be treated, all the way down to the papillae’s base. A full-thickness tunnel will be created, reaching supraperiosteal across the mucogingival edge into the alveolar mucosa. This is achieved by completely disclosing the tunnel-papillary network via a vertical incision and then through the gingival margins, permitting passive coronal substitution. This supraperiosteal tunnel shall be shifted coronally and adjusted inertly toward CEJ enveloping the recession faults. Coronal anchored sutures shall be inserted by involving the 2-3 mm apical gingival border of each tooth using a 4-0 silk suture. Once coronal stabilization is be achieved via the tunnel, a T-PRF clot will be uniformly distributed over recession defects. The mesial and distal sides of the treated teeth will be covered with a flowable composite material (without etching) to facilitate sutures. After thorough and appropriate adjustment of the graft, for the primary closure, the vertical incision will be sutured.

#### Surgical Procedure for the Control Group

##### Surgical Procedure for the Donor Area

Dentulous patients: To determine the size of the tissue graft, a periodontal probe is used for assessing the amount of GR. A tuberosity block will be performed along with a greater palatine nerve block. Two horizontal incisions will be made with a number 15 blade at the tuberosity area. The first incision will be 1-2 mm from the gingival margin. The second incision will be parallel and 4-5 mm away from the first incision. The third incision will be a connecting incision between first and second incision. After that the CTG of thickness 1-1.5 mm will be taken out with the surgical blade. The donor site will be pressed for 2-3 minutes with a wet gauze. The donor site will be closed by simple interrupted sutures with Vicryl (4-0) to control postoperative bleeding. No periodontal dressing will be placed due to possibility of displacement of the dressing.

Edentulous patients**:** A tuberosity block will be performed along with a greater palatine nerve block. Horizontal incision will be made on the edentulous tuberosity site with a number 15 blade. A vertical incision will be made at mesial aspect of the horizontal incision distal to the presenting tooth with a number 15 blade. A second vertical incision will be made at the distal aspect of the horizontal incision. The subepithelial flap will be raised and connective tissue will be taken out with surgical blade. The flap will be closed by simple interrupted sutures with Vicryl (4-0). No periodontal dressing will be placed due to possibility of displacement of the dressing.

Alternatively, the tuberosity area can also be negotiated for CTG harvest by de-epithelialization using a half round diamond bur and selection of a suitable diameter trephine bur [8].

##### Surgical Procedure for the Recipient Area

The surgical procedure for the control group will be the same as that for the test group procedure except that the tuberosity CTG will be placed using the M-VISTA technique on the recession defect area.

### Postoperative Care

Systemic antibiotics will be given. Patients shall be informed that the teeth should not be brushed in the treated areas for 3 weeks following the operation. For a duration of 14 days, all participants will be informed to use 0.2% chlorhexidine gluconate twice a day. The stitches will be removed after a period of 30 days. Improvement will now be visible. Polishing will be done after saline irrigation using polishing paste and a rubber cup with no traumatization to the healed area. Patients shall be advised to cleanse the managed site with cotton swab soaked in 0.2% chlorhexidine gluconate for the next 7 days and brush using Charter’s method. Follow-up with the patients will occur 1, 3, and 6 months after surgery.

### Statistical Analysis

Both univariate and multivariate analyses will be performed to evaluate the effect of various variables (such as baseline GT, WKG, and recession length and width) on the final outcomes. Means and SDs will be calculated using standard statistical procedures to ensure significance.

The data will be analyzed as follows:

1. Changes from baseline to 3 and 6 months will be assessed using a Student paired *t* test.
2. Comparisons between treatment groups at baseline, 3 months, and 6 months will be conducted using a Student unpaired *t* test.

In addition to these tests, multivariate analyses, such as multiple regression and analysis of covariance, will be used to determine the combined effects of covariates like baseline GT, WKG, and recession dimensions on treatment outcomes. These methods will allow for the assessment of potential interactions and the adjustment of confounding variables. The threshold for statistical significance will be set at *P*<.05.

## Results

The combination of the M-VISTA technique with T-PRF is expected to significantly enhance the efficiency and effectiveness of root coverage procedures. This approach is projected to deliver superior outcomes across key clinical parameters, including reductions in PPD and RD and improvements in CAL, relative attachment level, and RGML, as well as notable increases in the thickness of attached gingiva.

This study was initiated in July 2024 with a 3-month phase dedicated to recruitment, participant selection, baseline data collection, and randomization of groups, which concluded in September 2024. The intervention phase, which is currently underway (October 2024 to December 2025), focuses on performing T-PRF and CTG procedures for both groups using the M-VISTA technique. From January 2025 to June 2026, the follow-up phase will be conducted, involving monthly clinical assessments and monitoring of clinical parameters. Finally, the study will conclude in June 2026 with a 2-month phase for final evaluations, data analysis, comparison of results, and preparation for publication. This structured timeline ensures a systematic approach to achieving the study objectives. The study timeline is outlined in Table 2.

**Table 2:** Study timeline table.

Timeline	Study procedures	Duration
July 2024-Sept 2024	Recruitment, participant selection, baseline data collection, and randomization of groups	3 months
Oct 2024-Dec 2025	Intervention phase: T-PRF^a^ and CTG^b^ procedures for both groups using M-VISTA^c^ technique	15 months
Jan 2025-June 2026	Follow-up phase: monthly clinical assessments and monitoring of clinical parameters	18 months
June 2026-July 2026	Final evaluation, data analysis, comparison of results, conclusion of study, publication preparation	2 months

^a^T-PRF: titanium-prepared platelet-rich fibrin.

^b^CTG: connective tissue graft.

^c^M-VISTA: modified vestibular incision supraperiosteal tunnel access.

## Discussion

### Anticipated Findings

Based on existing evidence, we anticipate that the M-VISTA technique, when combined with either T-PRF or CTG, will be a viable approach for managing multiple GR defects. Previous studies have demonstrated that both techniques can significantly improve root coverage outcomes. T-PRF, in particular, has been shown to enhance soft tissue healing due to its autogenous nature, sustained release of growth factors, and ability to promote angiogenesis and fibroblast proliferation [14,15,19,20]. Given these documented benefits, T-PRF may serve as a potential alternative to CTG, aligning with current evidence-based trends in periodontal regeneration. Additionally, the findings align with previous literature, reinforcing the effectiveness of minimally invasive periodontal plastic surgery techniques in addressing GR defects.

Amin et al [12] in their split mouth clinical trial assessed the effects of donor and recipient locations and compared postoperative discomfort correlated with palatal and tuberosity donor areas for soft tissue transplantation. Compared with the palatal donor, the tuberosity donor’s pain level was significantly lower during the first 2 weeks after surgery. Whether the donors were palatal or tuberosity, the average proportion of root covering was the same (mean 67%, SD 12% vs mean 62%, SD 13%, respectively; *P*=102). According to the authors, soft tissues harvested from the tuberosity region may produce better outcomes than palatal function and reduce postoperative pain [12].

Since Allen introduced tunnelling in 1994 [6], several studies have been conducted on the process [16], despite the fact that many modifications to the approach have been suggested. VISTA is a minimally invasive procedure that was introduced by Zadeh [21]. Later, a version of this known as M-VISTA was described [8]. A case report by Chowdhary et al [9] described the M-VISTA method with CTG in the management of multiple Miller Class I or II buccal GR areas. A CTG was taken from the palate using the “trap door” technique and the vertical incision was sutured after the CTG was implanted in the supraperiosteal tunnel. According to the authors, total root coverage was attained and sustained at 9 months, with exceptional aesthetic results.

Fernandez-Jimenez et al [19] examined a case series in which the outcomes of the M-VISTA method were assessed 6 months later in patients with multiple GRs (Miller class III). After the intervention, the study’s mean root coverage was 58.72%, and 29% of the patients with GR had total root coverage. The authors came to the conclusion that M-VISTA might be superior to existing methods for class III recession in a number of ways [19]. In their work, Uzun et al [20] contrasted the treatment of several GRs with autogenous T-PRF and CTG. Keratinized tissue width (KTW), GT, RD, and clinical periodontal indices were recorded prior to surgery and at 6- and 12-month follow-up examinations. In addition, the values of the healing index and visual analogue scale were assessed. The study’s findings showed that, at a 12-month time point, the T-PRF and CTG groups had mean root coverages of 93.29% and 93.22%, respectively. In addition, the T-PRF and CTG groups had mean KTW increases of 1.97 mm and 0.75 mm, respectively. The treatment of several Miller Class I or II GR defects with T-PRF is both reliable and efficient. This has therapeutic significance since it suggests that T-PRF, an excellent autogenous substitute for CTG—the gold standard for root coverage—may be used [20].

Balice et al [22] published a study on 30 patients with 2 adjacent RT1 GRs treated using the coronally advanced flap (CAF) combined with either a CTG from the maxillary tuberosity (T-CTG, 15 patients) or leukocyte and platelet-rich fibrin (L-PRF). After 12 months, both techniques were effective in achieving root coverage and comparable patient-reported outcomes, but the CAF + T-CTG group showed significantly greater GT gain compared to the CAF + L-PRF group [22]. Matvijenko et al [23], in their systematic literature review, analyzed 5 RCTs comprising 195 cases of GRs to compare the tunnel technique and the M-VISTA method. The review revealed a significant increase in KTW (–1.4 mm), CAL (–2.65 mm), and RD (–2.7 mm) for the tunnel technique from baseline to 6 months. Conversely, the M-VISTA group showed a significant reduction in GR width (–2.26 mm). Both techniques were found effective in root coverage, with no significant differences in probing depths, highlighting their value for treating multiple GRs [23].

The above studies highlighted various advancements in treating GR but also presented limitations that warrant consideration. Many of the studies, such as those by Amin et al [12] and Balice et al [22], focused on short-term outcomes (6-12 months), leaving questions about the long-term stability of the results. The comparison of techniques, such as T-PRF versus CTG and M-VISTA versus the tunnel technique, often lacked uniformity in sample sizes, standardization of procedures, and follow-up periods, making it challenging to draw definitive conclusions. Additionally, some studies, like those by Fernandez-Jimenez et al [24] primarily addressed specific GR classifications (eg, Miller Class I and II or III), limiting the generalizability of findings to other types of recessions. Furthermore, while studies like that by Uzun et al [20] highlighted promising results with T-PRF, the reliance on advanced materials and techniques may pose practical and cost-related barriers in routine clinical settings. Lastly, patient-reported outcomes, such as pain and aesthetic satisfaction, were inconsistently reported across studies, suggesting a need for more comprehensive and standardized evaluation criteria to better understand the overall impact of these treatments. These limitations underscore the need for larger, multicenter, and long-term trials to validate the effectiveness and applicability of these approaches.

Despite the strengths of this randomized controlled trial, several limitations must be acknowledged. The relatively small sample size may limit the generalizability of the findings, necessitating larger multicenter studies to validate the results. While the follow-up period of 18 months provides valuable insights into the short- to mid-term outcomes, longer-term data are essential to assess the stability and sustainability of both techniques. The use of the Miller classification instead of the Cairo classification for GR assessment, though widely used in previous literature, does not account for interproximal attachment loss, potentially affecting prognostic accuracy. Additionally, while T-PRF eliminates the need for a second surgical site, thereby reducing patient morbidity and postoperative discomfort, its long-term regenerative potential compared to CTG, particularly in keratinized tissue augmentation, remains an area requiring further investigation. The study also does not incorporate patient-reported outcome measures, such as pain perception and aesthetic satisfaction, which are crucial factors in periodontal plastic surgery. Operator skill and variability in individual healing responses may further influence clinical outcomes. Future research should address these limitations by using larger sample sizes, extended follow-up durations, and standardized assessments to enhance the clinical applicability and long-term validation of these findings.

The Miller classification [25] was selected for this study due to its widespread clinical acceptance and established reliability in categorizing GR based on the likelihood of complete root coverage. It has been extensively used in periodontal research and provides a practical framework for surgical decision-making, particularly in cases where interproximal attachment loss is minimal or absent. In contrast, the Cairo classification [26] incorporates interproximal clinical attachment loss to distinguish between RT1, RT2, and RT3 recession types, making it more comprehensive for cases involving periodontal disease progression. However, the Miller classification remains highly relevant for evaluating root coverage procedures, as it directly correlates with treatment predictability and surgical outcomes.

Despite its advantages, the Miller classification has certain limitations. It does not account for interproximal bone levels, which can influence the prognosis of root coverage procedures, nor does it consider factors such as gingival biotype and tissue thickness, which are now recognized as critical determinants of treatment success. Additionally, its categorical nature may introduce variability in case classification among clinicians, potentially impacting reproducibility. Nonetheless, its extensive use in clinical studies allows for direct comparison with existing literature, ensuring consistency in evaluating periodontal treatment outcomes.

### Conclusions

The integration of T-PRF with the M-VISTA technique presents a promising advancement in periodontal therapy. By eliminating the need for a second surgical site, T-PRF serves as a less invasive alternative to CTG, potentially reducing patient morbidity and enhancing postoperative recovery. This study highlights the potential for significant improvements in periodontal parameters and soft tissue outcomes within a shorter regeneration period. Additionally, this approach addresses the challenges associated with GR management while optimizing treatment efficiency and patient comfort. The findings emphasize the importance of incorporating biomaterials and minimally invasive surgical techniques in periodontal care. Further research is warranted to evaluate the long-term stability and broader clinical applications of this combined technique.

## Abbreviations

CAF: coronally advanced flap
CAL: clinical attachment level
CEJ: cementoenamel junction
CTG: connective tissue graft
GR: gingival recession
GT: gingival thickness
KTW: keratinized tissue width
M-VISTA: modified vestibular incision supraperiosteal tunnel access
PI: plaque index
PPD: pocket probing depth
PRF: platelet-rich fibrin
RAL: relative attachment level
RC: root coverage
RD: recession depth
RGML: relative gingival margin level
T-PRF: titanium-prepared platelet-rich fibrin
VISTA: vestibular incision supraperiosteal tunnelling access
WKG: width of keratinized gingiva

## Data Availability

The data supporting the findings of this study are available from the corresponding author upon reasonable request. Due to ethical considerations and institutional regulations, certain restrictions may apply to data sharing.

## References

[1] Kao RT, Curtis DA, Kim DM, Lin G, Wang C, Cobb CM, Hsu Y, Kan J, Velasquez D, Avila‐Ortiz G, Yu S, Mandelaris GA, Rosen PS, Evans M, Gunsolley J, Goss K, Ambruster J, Wang H (2020). American Academy of Periodontology best evidence consensus statement on modifying periodontal phenotype in preparation for orthodontic and restorative treatment. J Periodontol.

[2] Fujita T, Yamamoto S, Ota M, Shibukawa Y, Yamada S (2011). Coverage of gingival recession defects using guided tissue regeneration with and without adjunctive enamel matrix derivative in a dog model. Int J Periodontics Restorative Dent.

[3] Merijohn GK (2016). Management and prevention of gingival recession. Periodontol 2000.

[4] Jung U, Um Y, Choi S (2008). Histologic observation of soft tissue acquired from maxillary tuberosity area for root coverage. J Periodontol.

[5] Raetzke PB (1985). Covering localized areas of root exposure employing the "envelope" technique. J Periodontol.

[6] Allen AL (1994). Use of the supraperiosteal envelope in soft tissue grafting for root coverage. I. Rationale and technique. Int J Periodontics Restorative Dent.

[7] Zabalegui I, Sicilia A, Cambra J, Gil J, Sanz M (1999). Treatment of multiple adjacent gingival recessions with the tunnel subepithelial connective tissue graft: a clinical report. Int J Periodontics Restorative Dent.

[8] Zadeh HH (2011). Minimally invasive treatment of maxillary anterior gingival recession defects by vestibular incision subperiosteal tunnel access and platelet-derived growth factor BB. Int J Periodontics Restorative Dent.

[9] Chowdary PC, Pavan Kumar YS, Murthy KRV, Kishore DT (2022). A novel modified-vista technique with connective tissue graft in the treatment of gingival recession: a case report. Clin Adv Periodontics.

[10] Tolstunov L (2009). Maxillary tuberosity block bone graft: innovative technique and case report. J Oral Maxillofac Surg.

[11] Younes R, Khairallah C (2020). The "one piece" autologous tuberosity graft: a contemporary concept in ridge preservation. Case Rep Dent.

[12] Amin PN, Bissada NF, Ricchetti PA, Silva APB, Demko CA (2018). Tuberosity versus palatal donor sites for soft tissue grafting: a split-mouth clinical study. Quintessence Int.

[13] Konflanz W, Orth CC, Celeste RK, Muniz FWMG, Haas AN (2021). Influence of donor site and harvesting technique of connective tissue graft on root coverage putcomes of single gingival recessions: systematic review and meta-analyses. J Int Acad Periodontol.

[14] Tunali M, Ozdemir H, Kucukodaci Z, Ezirganli S, Baris E, Akman S, Atay A, Firatli E (2015). A novel platelet concentrate for guided bone regeneration: titanium prepared platelet-rich fibrin (T-PRF). Gulhane Med J.

[15] Petrungaro PS (2001). Using platelet-rich plasma to accelerate soft tissue maturation in esthetic periodontal surgery. Compend Contin Educ Dent.

[16] Collins JR, Cruz A, Concepción E, López C, Hou W, Romanos GE (2021). Connective tissue graft vs rlatelet-rich fibrin in the treatment of gingival recessions: a randomized split-mouth case series. J Contemp Dent Pract.

[17] Naruke T, Yoneyama T, Shimosato Y (1970). Case of primary osteogenic sarcoma of the lung. Gan No Rinsho.

[18] Isenberg HD, Berkman JI (1971). Prevalence of extrachromosomal drug resistance. The role of drug-resistant and drug-selected bacteria in nosocomial disease. Ann N Y Acad Sci.

[19] Fernández-Jiménez A, Estefanía-Fresco R, García-De-La-Fuente AM, Marichalar-Mendia X, Aguirre-Zorzano L (2021). Description of the modified vestibular incision subperiosteal tunnel access (m-VISTA) technique in the treatment of multiple Miller class III gingival recessions: a case series. BMC Oral Health.

[20] Uzun BC, Ercan E, Tunalı M (2018). Effectiveness and predictability of titanium-prepared platelet-rich fibrin for the management of multiple gingival recessions. Clin Oral Investig.

[21] Zadeh HH (2011). Minimally invasive treatment of maxillary anterior gingival recession defects by vestibular incision subperiosteal tunnel access and platelet-derived growth factor BB. Int J Periodontics Restorative Dent.

[22] Balice G, Paolantonio M, Serroni M, De Ninis P, Rexhepi I, Frisone A, Di Gregorio S, Romano L, Sinjari B, Murmura G, Femminella B (2024). Treatment of multiple RT1 gingival recessions using a coronally advanced flap associated with L-PRF or subgingival connective tissue graft from maxillary tuberosity: a randomized, controlled clinical trial. Dent J (Basel).

[23] Matvijenko K, Borusevičius R (2024). Comparison of tunnel and VISTA techniques for multiple gingival recession treatment: a systematic literature review. J Adv Periodontol Implant Dent.

[24] Fernández-Jiménez A, Estefanía-Fresco R, García-De-La-Fuente AM, Marichalar-Mendia X, Aguirre-Urizar JM, Aguirre-Zorzano LA (2023). Comparative study of the modified VISTA technique (m-VISTA) versus the coronally advanced flap (CAF) in the treatment of multiple Miller class III/RT2 recessions: a randomized clinical trial. Clin Oral Investig.

[25] Miller PD (1985). A classification of marginal tissue recession. Int J Periodontics Restorative Dent.

[26] Cairo F, Nieri M, Cincinelli S, Mervelt J, Pagliaro U (2011). The interproximal clinical attachment level to classify gingival recessions and predict root coverage outcomes: an explorative and reliability study: interproximal CAL for gingival recessions. J Clin Periodontol.

